# Maternal Gestational Hypertension Is Associated with Reduced TSG-6 in Human Milk: Potential Implications for Neonatal Immunological Support

**DOI:** 10.3390/ijms27198573

**Published:** 2026-09-25

**Authors:** Dominika Przywara, Adrianna Kondracka, Alicja Petniak, Patrycja Słyk-Gulewska, Magdalena Drąg, Urszula Szymanowska, Monika Czuba, Milena Jaremek, Aneta Strachecka, Janusz Kocki, Paulina Gil-Kulik

**Affiliations:** 1 Department of Clinical Genetics, Medical University of Lublin, 11 Radziwillowska Str., 20-080 Lublin, Poland; dprzywara17@gmail.com (D.P.); alicja.petniak@umlub.pl (A.P.); monikaczuba@umlub.pl (M.C.); janusz.kocki@umlub.pl (J.K.); 2 Doctoral School, Medical University of Lublin, 20-093 Lublin, Poland; 3 Department of Obstetrics and Pathology of Pregnancy, Medical University of Lublin, 11 Staszica Str., 20-081 Lublin, Poland; adrianna.kondracka@umlub.pl (A.K.); slyk.patrycja@gmail.com (P.S.-G.); magdalena.drg@gmail.com (M.D.); 4 Department of Biochemistry and Food Chemistry, University of Life Sciences, 8 Skromna Str., 20-704 Lublin, Poland; urszula.szymanowska@up.lublin.pl; 5 Department of Invertebrate Ecophysiology and Experimental Biology, Faculty of Environmental Biology, University of Life Sciences in Lublin, 54 Doświadczalna Street, 20-280 Lublin, Poland; milena.jaremek@up.edu.pl (M.J.); aneta.strachecka@up.edu.pl (A.S.)

**Keywords:** human milk mesenchymal stem cells, HM-MSCs, TSG-6, gestational hypertension, microRNA, fetal metabolic status

## Abstract

Tumor necrosis factor alpha-stimulated protein 6 (TSG-6) is a potent anti-inflammatory mediator secreted by mesenchymal stem cells (MSCs). In human milk (HM), these cells (human milk mesenchymal stem cells, HM-MSCs) contribute to postnatal programming of the neonatal gastrointestinal immune axis. This study assessed how maternal gestational hypertension and maternal age affect the cytoprotective TSG-6 pool and its associated immunomodulatory microRNA signature in early milk. Early colostrum, 2–4 days postpartum, from healthy mothers and hypertensive patients was analyzed. *TSG-6* gene expression in the heterogeneous milk cellular fraction containing HM-MSCs and TSG-6 protein concentration in whole milk were determined using quantitative polymerase chain reaction (qPCR) and Enzyme-Linked Immunosorbent Assay (ELISA), respectively. Comprehensive multivariable General Linear Models (GLMs) were used to adjust for potential confounding variables, including pre-pregnancy Body Mass Index (BMI), gestational age (GA), and gestational diabetes. Additionally, TSG-6 levels were correlated with fetal cord blood gas parameters and previously profiled microRNAs in the cellular fraction. GLM analysis revealed that gestational hypertension remained significantly associated with reduced *TSG-6* gene expression in the cellular fraction and total TSG-6 protein concentration in whole milk, regardless of pre-pregnancy BMI or metabolic comorbidities. In healthy pregnancies, exploratory analyses suggested that TSG-6 levels correlated positively with optimal fetal metabolic status (cord blood pH). Importantly, *TSG-6* expression showed a strong positive correlation with a signature of immunomodulatory microRNAs (miR) (miR-155-5p, miR-21-3p, miR-126-3p). These microRNAs were concurrently downregulated in the hypertensive group; however, because all hypertensive participants received methyldopa, the independent contributions of hypertension and antihypertensive treatment to these changes cannot be distinguished. Gestational hypertension independently reduces the anti-inflammatory TSG-6 in early breast milk. The concomitant reduction in TSG-6 and its correlated microRNA co-expression pattern indicates an altered immunomodulatory profile within the mammary gland during lactation. We hypothesize that these changes might alter the local immunomodulatory properties of milk in the neonatal gastrointestinal tract. However, because no functional in vitro or in vivo assays were performed, this remains a purely theoretical implication that requires future experimental validation.

## 1. Introduction

Hypertension is a condition in which blood pressure exceeds 140/90 mmHg [1]. This condition is characterized by an inflammatory basis, which is related to the fact that chronic hypertension leads to endothelial damage, which in turn can initiate a cascade of immune reactions [2]. Reducing the activity of immune cells, including T lymphocytes, monocytes, and macrophages, has been shown to inhibit the development of hypertension [2,3].

Due to the development of inflammation and activation of immune cells in hypertension, increased secretion of proinflammatory cytokines, such as IL-1β, IL-17A, TNF-α, and IFN-γ, is observed [3,4]. These cytokines, in turn, stimulate the production of TSG-6 protein. *TSG-6* expression increases under inflammatory conditions, which allows it to exert a protective effect on tissues. This protein limits the activity of neutrophils, T lymphocytes, macrophages, and other immune cells [5]. Furthermore, TSG-6 inhibits the proinflammatory TLR2/MyD88/NF-κB signaling pathway, thereby reducing the production of proinflammatory cytokines [6].

Additionally, TSG-6 has the ability to bind hyaluronic acid (HA) [7]. The biological activity of HA depends on its structure. In its high-molecular-weight form (HMW) (>800 kDa), as well as in complexes formed in reactions catalyzed by TSG-6, HA exhibits anti-inflammatory and antioxidant properties. Furthermore, HA participates in the organization of the extracellular matrix (ECM). In combination with TSG-6, hyaluronic acid forms a stable and compact ECM structure, whereas in the absence of TSG-6, the matrix becomes highly hydrated and consequently unstable [8]. Fragmentation of HA, which can occur, among other things, as a result of vascular stress in hypertension, exerts a strong pro-inflammatory effect (Figure 1) [9]. In contrast, low- and medium-molecular-weight HA (LMW) (10–300 kDa and 300–800 kDa, respectively) stimulates angiogenesis, cell growth, and tissue regeneration [10].

In inflammatory conditions, TSG-6 is secreted by, among others, mesenchymal stem cells (MSCs). These cells have broad paracrine properties and are capable of accelerating the regeneration of damaged tissues and reducing inflammation [11]. TSG-6 has been identified as a key mediator responsible for the anti-inflammatory properties of MSCs [12], which has been used in experimental therapies for various inflammatory diseases, including pneumonia, kidney disease, intervertebral disc disease, and neuroinflammation [13,14,15,16].

MSCs are present in most human tissues [17], including human milk (HM-MSCs). HM-MSCs have been shown to penetrate the infant’s gastrointestinal tract during breastfeeding, from where they can migrate and integrate with the structures of the developing organs of the newborn [18,19]. Hyaluronic acid is also naturally present in human milk [20]. Beyond serving merely as a nutritional barrier, this cellular fraction functions as a dynamic biochemical communication system. Their trophic potential—including the secretion of anti-inflammatory TSG-6—is hypothesized to contribute to the postnatal development of the newborn’s immune axis and intestinal barriers. Human milk is not only a source of nutrients but also a complex biological fluid facilitating intergenerational communication, and microRNAs are becoming key, multifaceted regulators of infant health and development [21,22].

Although the impact of pregnancy pathologies on fetal development is studied within the DOHaD (Developmental Origins of Health and Disease) paradigm, the impact of chronic vascular stress (hypertension) on the immunomodulatory properties of colostrum remains poorly understood. Previous studies on umbilical cord stem cells (UC-MSCs) have not demonstrated a direct correlation between *TSG-6* expression and the occurrence of hypertension in pregnant women [23], but have demonstrated a strong correlation between *TSG-6* expression and cord blood gas parameters. The presence of such adaptive mechanisms underscores the need for a deeper understanding of how milk stem cells respond to maternal pathology and fetal metabolic status.

In light of the above reports, the aim of this study was to assess the effect of maternal gestational hypertension on *TSG-6* gene expression and protein concentration in colostrum, while taking into account the metabolic status of the newborn (cord blood gas parameters: pH, lactate (LAC), and base excess (BE)). To reliably assess the significance of vascular stress and exclude the influence of potential confounding variables (such as maternal age, BMI, or gestational age), GLMs were used. It is expected that these results will help answer the question of whether maternal gestational hypertension is independently associated with reduced *TSG-6* expression in the heterogeneous human milk cellular fraction and TSG-6 protein in early milk.

## 2. Results

### 2.1. Assessment of MSCs Presence Based on the Immunophenotyping and Cell Morphology

Flow cytometric analysis of the heterogeneous human milk cellular fraction revealed a distinct subpopulation (accounting for approximately 45% of the analyzed events) that robustly expressed the mesenchymal surface antigens CD73, CD90, CD105, and CD146, while lacking the hematopoietic and endothelial markers CD45, CD31, CD19, and CD14. This profile is consistent with the immunophenotype characteristic of HM-MSCs (representative flow-cytometry plots are provided in Supplementary Figure S1). Moreover, microscopic evaluation confirmed morphology typical of MSCs. The cells exhibited a spindle-shaped morphology with a single nucleus (Figure 2).

### 2.2. Assessment of TSG-6 Expression at the Transcript and Protein Levels

Based on the conducted study, *TSG-6* gene expression at the transcript level was detected by qPCR in 48 samples of the cellular fraction of early HM, and TSG-6 protein levels were assessed by ELISA in 71 samples of whole early HM. Importantly, as TSG-6 protein was measured in whole milk, we cannot determine whether the detected TSG-6 originated from HM-MSCs or from other cell types present in the milk.

The analyses demonstrated a statistically significant positive correlation between *TSG-6* gene expression in the human milk cellular fraction and TSG-6 protein concentration in whole HM (r = 0.479, *p* < 0.05; Figure 3).

Further analyses evaluated the influence of available clinical parameters related to maternal health, the course of pregnancy and delivery, and neonatal condition on *TSG-6* gene expression and protein concentration. The analyses revealed significantly lower *TSG-6* expression and significantly reduced TSG-6 protein levels in HM derived from women with gestational hypertension (Table 1, Figure 4). The concordant reduction in *TSG-6* gene expression and protein concentration is the strongest aspect of the study. However, it should be noted that all women with hypertension were concomitantly treated with methyldopa, therefore, an association between TSG-6 levels and antihypertensive treatment cannot be excluded.

To rigorously confirm our findings while addressing potential confounding factors, comprehensive multivariable General Linear Models (GLMs) were performed (Table 2). To address differences between the study groups and test the robustness of the associations, the models included maternal age, pre-pregnancy BMI, gestational age (GA), and the presence of gestational diabetes as covariates. In the first model (adjusted R^2^ = 0.232, *p* = 0.010), gestational hypertension remained significantly associated with reduced *TSG-6* gene expression (F = 7.337, *p* = 0.010, ηp^2^ = 0.166, ß = 0.446, 95% CI: 0.112 to 0.780). Gestational age, pre-pregnancy BMI, maternal age, and gestational diabetes did not show significant effects.

Crucially, parallel GLM analysis for TSG-6 protein concentration corroborated these transcriptomic shifts. Even after adjusting for the aforementioned covariates, gestational hypertension remained significantly associated with reduced TSG-6 protein levels (F = 4.276, *p* = 0.043, ηp^2^ = 0.063, ß = 2.604, 95% CI: 0.088 to 5.121). These results demonstrate that the reduction in TSG-6 is strongly associated with the gestational hypertension phenotype, irrespective of gestational age differences or metabolic comorbidities. In both models, with the normotensive control group serving as the reference category, the adjusted effect estimates confirmed the direction of the change: TSG-6 levels were significantly lower in the gestational hypertension group.

Furthermore, correlations between *TSG-6* gene expression and TSG-6 protein concentration and parameters characterizing the course of pregnancy, maternal health status, and neonatal condition were analyzed in both groups. The results are presented in Table 3.

In the control group, exploratory analyses revealed a nominally significant positive correlation between TSG-6 protein concentration in HM and umbilical cord blood pH (r = 0.481, *p* = 0.0268, q > 0.05) (Figure 5A), as well as a positive correlation between *TSG-6* gene expression (logRQ) and umbilical cord blood base excess (BE) (r = 0.467, *p* = 0.0255, q > 0.05) (Figure 5B). A significant negative correlation was found between logRQ *TSG-6* and venous umbilical cord blood lactate (LAC) levels (r = −0.529, *p* = 0.0276, q > 0.05) (Figure 5C) as well as between TSG-6 protein concentration in HM and venous umbilical cord blood LAC (r = −0.387, *p* = 0.0144, q > 0.05) (Figure 5D).

Additionally, a moderate positive correlation was demonstrated between logRQ *TSG-6* and neonatal birth weight (r = 0.385, *p* = 0.0374, q > 0.05) (Figure 6A), and a negative correlation between logRQ *TSG-6* and maternal age (r = −0.391, *p* = 0.0138, q > 0.05) (Figure 6B). No other statistically significant associations were observed between logRQ *TSG-6* or TSG-6 protein concentration and the analyzed parameters (*p* > 0.05, Table 3).

Due to the limited sample size of the hypertensive cohort, the associations observed in the control group did not reach statistical significance in the hypertensive group (*p* > 0.05, Table 3).

Due to the high number of comparisons, original *p*-values were subjected to Benjamini–Hochberg FDR correction. Only correlations between *TSG-6* gene expression and miR (miR-21-3p, miR-155-5p, miR-126-3p) remained statistically significant after FDR adjustment (q < 0.05).

Finally, we examined the association between *TSG-6* transcript levels and a panel of microRNAs (miR-155-5p, miR-21-3p, and miR-126-3p) previously profiled in the same cohort [24]. Interestingly, our analysis revealed strong and statistically significant positive correlations between *TSG-6* gene expression and all assessed miR (miR-21-3p r = 0.725, *p* < 0.0000001, q < 0.05 **; miR-126-3p r = 0.579, *p* = 0.0006, q < 0.05 **; miR-155-5p r = 0.783, * *p* < 0.000001, q < 0.05 **) (Table 3, Figure 7A–C).

## 3. Discussion

To the best of our knowledge, this is the first report describing the association between maternal gestational hypertension and *TSG-6* expression in the heterogeneous human milk cellular fraction containing an MSC-like population. TSG-6 is a key anti-inflammatory mediator that plays a significant role in hyaluronic acid binding and extracellular matrix organization [3,4,5,6,7,8,9,10]. Previous studies have shown that impaired maternal blood pressure regulation affects the composition of human milk, including the levels of proteins, lipids, carbohydrates, and growth factors such as NGF and BDNF [25].

To the best of our knowledge, there are no studies clearly defining the association between hypertension and the immunomodulatory properties of human milk. However, this phenomenon has been widely described in the context of obesity, a condition that often coexists with vascular pathology. Maternal obesity has been associated with decreased antibody levels (IgG, IgM, and IgA) and increased levels of proinflammatory cytokines, including IL-6, IL-10, IL-4, and TNF-α, in early milk, resulting in a distinct proinflammatory profile [26]. We therefore used multivariable GLM analysis to account for potential metabolic confounding.

Our GLM analysis showed that gestational hypertension was independently associated with reduced *TSG-6* gene expression in the human milk cellular fraction and lower TSG-6 protein concentrations in whole milk. This association remained statistically significant after adjustment for potential confounding variables, including maternal age, pre-pregnancy BMI, gestational age, and gestational diabetes. These findings suggest that the association between maternal gestational hypertension and TSG-6 levels cannot be fully explained by differences in body weight or gestational duration.

Importantly, these findings should be interpreted in the context of the clinical management of the patients. Since all women in the hypertensive group received standard treatment with methyldopa to control blood pressure, the study design does not allow us to completely distinguish the specific molecular consequences of the underlying vascular pathology from the potential effects of the medication. Therefore, the observed reduction in *TSG-6* expression and TSG-6 protein concentration reflects the in vivo state of the mammary gland resulting from the combined effects of gestational hypertension and the necessary pharmacological treatment.

Our study also demonstrated a positive correlation between *TSG-6* gene expression in the cellular fraction and TSG-6 protein concentration in whole milk, consistent with findings reported in other tissues [27].

It should be noted, however, that whole milk is a complex biological fluid. Although we observed a parallel reduction in *TSG-6* transcript levels in the isolated cellular fraction, which was enriched in HM-MSCs, and in TSG-6 protein levels in whole milk, ELISA measurements do not allow the specific cellular source of this protein to be identified. TSG-6 protein in whole milk likely originates from multiple sources, including mammary epithelial cells, various milk leukocytes, and potentially from plasma transudate. Therefore, the total TSG-6 pool in milk cannot be attributed exclusively to secretion by HM-MSCs, although the concordant reduction in *TSG-6* gene expression in these cells suggests that they may represent an important component of this system affected by the observed changes.

Human milk serves not only nutritional purposes but also constitutes a dynamic biochemical communication system, delivering immunomodulatory factors and stem cells directly to the newborn’s gastrointestinal tract. This process may contribute to postnatal development of the immune system and maturation of the intestinal barrier. While the influence of neonatal clinical status on the overall immunological composition of human milk has been previously described [25], reduced TSG-6 levels in milk from mothers with hypertension have not been previously reported, making this finding particularly noteworthy. In a mouse model, Romano et al. demonstrated that MSCs deficient in TSG-6 exhibited reduced proliferative and differentiation potential and secreted higher levels of proinflammatory cytokines [28,29]. Lauer et al. reported increased *TSG-6* expression in lung tissue from patients with idiopathic pulmonary arterial hypertension [30]. During breastfeeding, the cellular fraction containing HM-MSCs is transferred to the infant, where it may interact with neonatal tissues [18,19].

Because no functional in vitro or in vivo assays were performed in this study, the direct implications of reduced TSG-6 for neonatal gastrointestinal immune protection remain entirely speculative. We hypothesize that lower TSG-6 levels might theoretically alter the local anti-inflammatory capacity of human milk in the immature gut. However, our purely observational data do not provide direct evidence that this phenomenon clinically affects intestinal health or the pathophysiology of conditions such as necrotizing enterocolitis (NEC) in newborns.

By catalyzing the formation of high-molecular-weight complexes with HA, TSG-6 also participates in the structural stabilization of the extracellular matrix (ECM). Vascular stress has previously been associated with increased HA degradation, and HA fragmentation may stimulate the expression of antibacterial defenses and promote inflammation [9,20]. Ormiston et al. demonstrated that pulmonary hypertension may enhance HA degradation, potentially affecting ECM organization [9]. Fragmented HA has been shown to act as a signal of tissue damage and increase the expression of antibacterial defenses [20]. In addition, low-molecular-weight HA may support intestinal maturation [10]. However, Sammarco et al. demonstrated that HA contributes to intestinal epithelial regeneration in the presence of TSG-6 [7]. Interestingly, the neonatal intestine’s supply of TSG-6 may depend not only on direct ingestion but also on local synthesis, for instance, high levels of breast milk polyamines have been shown to upregulate *TSG-6* gene expression in infant gut cells, potentially enhancing extracellular matrix organization and gut maturation [31].

The reduced TSG-6 levels in colostrum from hypertensive mothers may be consistent with alterations in structural homeostasis within the mammary gland. Importantly, this reduction in the stabilizing protein TSG-6 is consistent with our recent transcriptomic data, which showed that maternal gestational hypertension was associated with substantial changes in the transcriptional profile of perinatal stem cells. Gene ontology (GO) analysis revealed enrichment of pathways related to extracellular matrix remodeling and activation of the canonical Wnt pathway in these cells [32]. Therefore, the reduction in anti-inflammatory TSG-6 levels in whole milk may not represent an isolated finding but could be part of broader hypertension-associated alterations in the maternal cellular niche, including processes related to fibrosis and extracellular matrix disorganization. Consequently, human milk may have reduced regenerative potential, and the regeneration of the neonatal intestinal epithelium, which may involve TSG-6-dependent interactions with HA [7], could potentially be affected.

Our findings, together with evidence from the literature, raise the possibility that reduced TSG-6 levels associated with maternal gestational hypertension may influence neonatal intestinal regeneration, immune protection, the TSG-6/HA axis, and the putative mother–milk–neonate communication axis. It should be noted, however, that this remains a hypothesis that requires confirmation in further research. The associations identified in our study are limited to *TSG-6* gene expression and TSG-6 protein concentrations. Importantly, no functional assays were performed in the present study, such assays are necessary to test the proposed hypothesis and establish the biological relevance of the observed associations.

The overall reduction in TSG-6 levels in the context of maternal gestational hypertension is not an isolated finding but may be part of a broader pattern of alterations in perinatal cellular niches. Our previous studies demonstrated that gestational hypertension is associated with reduced expression of key immunomodulatory microRNAs in the milk cellular fraction, including miR-155, miR-126, miR-21, and miR-29a [24]. As we recently reported, this phenomenon was associated with alterations in the trophic profile of HM-MSCs, including decreased *HGF* and *IGF1* transcript levels, which in turn were closely correlated with miR-155 levels [32]. Importantly, in the current analysis, we observed a strong positive correlation between *TSG-6* gene expression and the levels of key microRNAs, including miR-155-5p, miR-21-3p, and miR-126-3p, in the milk cellular fraction.

miR-155-5p and miR-21 have been reported to participate in the regulation of immunomodulatory processes [33,34], whereas miR-126 is recognized as an important regulator of endothelial homeostasis and angiogenesis [35]. It is worth emphasizing that the importance of miR-155 extends beyond local regulation in the mammary gland. This molecule has been implicated in the pathogenesis of arterial hypertension [36] and has been proposed as a potential non-invasive marker of target organ damage in essential hypertension [37]. Therefore, the positive correlations observed between *TSG-6* expression and the levels of miR-155-5p, miR-21-3p, and miR-126-3p may reflect coordinated regulation of pathways involved in immune response modulation, vascular homeostasis, and tissue repair. Importantly, these findings should be interpreted in the context of the reduced *TSG-6* expression observed in the HM cellular fraction derived from mothers with gestational hypertension. Together with our previous observations of decreased levels of immunomodulatory microRNAs in the milk cellular fraction, these results suggest that gestational hypertension may be associated with alterations in the correlated expression pattern of immunomodulatory miR in this heterogeneous cell population, which may be relevant to its immunoregulatory and reparative properties [24].

The observed coexpression between *TSG-6* and the analyzed microRNAs may indicate that these molecules are associated with shared vascular and immune-related pathways in colostrum-derived cells. The simultaneous reduction in TSG-6 and its associated co-expression pattern of posttranscriptional regulators suggests that the anti-inflammatory molecular profile of early milk may be altered in pregnancies complicated by maternal vascular pathology. The observed changes in this correlative molecular module may reflect alterations in maternal cellular adaptive responses associated with the hypertensive state and its treatment.

Moreover, our study is among the first to examine the relationship between umbilical cord blood gasometric parameters (pH, LAC, and BE) and TSG-6 levels in colostrum. In the healthy cohort, we observed a positive correlation between TSG-6 protein concentration and cord blood pH and a negative correlation with LAC. These associations may reflect a relationship between TSG-6 levels in human milk and the metabolic and acid–base status of the newborn, consistent with our previous observations in a model of umbilical cord-derived mesenchymal stem cells (UC-MSCs) [23]. We previously demonstrated that physiological pH increases *TSG-6* gene expression [23], whereas metabolic stress and acidosis reduce the expression of several regenerative factors, including Epidermal Growth Factor (*EGF*), Fibroblast Growth Factor Receptor 1 (*FGFR1*), Nerve Growth Factor (*NGF*), and Insulin-like Growth Factor 1 (*IGF1*). It should be emphasized, however, that the analyses were exploratory in nature [38].

In the case of gestational hypertension, we observed an absolute reduction in TSG-6 concentrations. Instead of physiological adaptation to the needs of the fetus, chronic vascular pathology appears to induce a state that we interpret as potential depletion of local anti-inflammatory reserves within the mammary gland. We also observed a strong negative correlation between logRQ *TSG-6* and umbilical cord blood LAC levels, as well as between TSG-6 protein concentration and LAC. Elevated LAC levels may reflect increased anaerobic metabolism and, in some clinical contexts, hypoxia, and are often associated with lower pH values. Therefore, the negative correlations between TSG-6 and LAC, together with the positive correlation between TSG-6 and pH, are mutually consistent.

The exploratory correlations observed in healthy women between TSG-6 expression and umbilical cord blood LAC, pH, and BE suggest an association between the anti-inflammatory profile of early human milk and neonatal metabolic status. These findings indicate that a more favorable acid–base balance (lower lactate levels, higher pH and BE) is associated with higher TSG-6 concentrations in human milk. However, while these results are mutually consistent, they do not establish that neonatal metabolic status directly regulates the immunological composition of maternal milk.

Similar trends were observed in the milk of women with gestational hypertension; however, the smaller sample size in this group precluded statistical significance. The apparent absence of correlations with blood gas parameters in the hypertensive group should therefore be interpreted with caution. The small sample size limits the statistical power to reliably assess these associations and formally test interactions within this subgroup. Accordingly, our interpretation of this finding is primarily based on the observed reduction in the overall TSG-6 protein concentration.

While the correlations between *TSG-6* and umbilical cord blood gas parameters (pH, LAC, BE) showed nominally significant original *p*-values (*p* < 0.05), they did not survive the rigorous FDR correction (q > 0.05). Therefore, these specific associations should be treated as exploratory, hypothesis-generating observations suggesting a potential link between fetal metabolic stress and colostrum immunological composition, which warrants confirmation in larger, targeted cohorts.

Another aspect analyzed in our study was the relationship between *TSG-6* and clinical parameters and available laboratory test results. Although higher CRP levels were observed in hypertensive mothers than in normotensive mothers, no association was found between TSG-6 concentration and CRP levels. This contrasts with the acute inflammatory response observed, for example, during early infections, as described by Tomaszewska et al., in which milk cells respond dynamically [39]. These findings suggest that the reduction in TSG-6 levels in colostrum may not be primarily associated with acute systemic inflammation but could reflect longer-term alterations associated with maternal gestational hypertension.

We also identified a moderate positive correlation between *TSG-6* expression and neonatal birth weight. We have previously described an association between birth weight and the expression of *IL1B* and *IL6R* in perinatal stem cells, genes that may be functionally related to pathways involved in TSG-6 activity [23]. The reduced TSG-6 levels observed in the hypertensive group may therefore be considered in the context of the association between maternal gestational hypertension and lower birth weight and neonatal maturity [40,41].

### Strengths and Limitations

When interpreting the results of this study, several important limitations should be considered. The main limitation is the relatively small sample size of patients with hypertension (N = 9), which naturally weakens the statistical power to assess intra- and inter-correlation interactions in this subpopulation. However, to minimize this risk and reliably assess the impact of gestational hypertension, we used rigorous multivariate linear models. These models confirmed that the observed decrease in *TSG-6* is a robust phenomenon independent of other clinical variables.

Second, due to absolute clinical safety requirements, all nine women in the hypertensive group received methyldopa treatment. Consequently, in the study cohort, antihypertensive treatment was completely confounded with the presence of gestational hypertension itself. The direct and independent effects of this pharmacotherapy on the colostrum transcriptome and the secretory profile of this cellular fraction represent an important potential confounding factor. Therefore, it is possible that the observed molecular alterations were partly attributable to the effects of the medication rather than exclusively to vascular stress. Distinguishing the independent effects of the disease from those of the medication will require targeted in vitro models or studies in larger clinical cohorts that account for variation in the type and dose of antihypertensive medications used.

Another limitation of the study is that the analysis was limited to early HM collected between days 2 and 4 postpartum. Given the dynamic changes in milk composition during lactation, the results cannot be directly extrapolated to transitional or mature milk. The study is also observational in nature—the changes demonstrated at the mRNA and total protein levels describe the cellular phenotype in vivo, but do not allow for firm conclusions regarding cause and effect. Possible confirmation of how reduced TSG-6 levels physically impact infant gastrointestinal health will require advanced functional studies in vitro and close, long-term clinical follow-up of the children.

Furthermore, because TSG-6 protein levels were assessed in whole milk rather than using targeted in vitro secretion assays, the precise proportional contribution of HM-MSCs to the total TSG-6 protein pool in human milk remains to be quantitatively determined in functional studies.

The undeniable strength of the study is that it is one of the first to combine cellular assessment of colostrum fractions with proteomic analysis of whole milk, enriched with precise maternal clinical background and neonatal blood gas status, opening new perspectives in understanding maternal immune programming. However, due to the observational nature of the study, the identified correlations do not allow for definitive conclusions regarding cause and effect relationships. The results should rather be interpreted as evidence of the coexistence of metabolic and immunological phenomena.

## 4. Materials and Methods

### 4.1. Study Material

The study material consisted of the heterogeneous human milk cellular fraction and whole HM obtained from women hospitalized at the Department of Obstetrics and Pregnancy Pathology, University Clinical Hospital No. 1 in Lublin. The cellular fraction was used to assess *TSG-6* gene expression whereas whole milk samples were used to determine TSG-6 protein concentrations.

Milk samples were collected 2–4 days postpartum. Each participant was informed of the purpose of the study and provided written, informed consent. The study was conducted after obtaining approval from the Bioethics Committee (Resolution No. KE-0254/7/01/2023; approval date 26 January 2023).

*TSG-6* gene expression was assessed in milk cell fraction samples obtained from 48 women, whereas TSG-6 protein concentrations were measured in whole milk samples collected from 71 women. The difference in sample size was due to limitations in the volume of biological material available. In some participants, an insufficient volume of early milk was obtained, precluding the isolation of a sufficient number of cells required for high-quality RNA extraction.

Among the women studied, a subpopulation of patients diagnosed with gestational hypertension (GH) was identified, constituting the study group (N = 9). Gestational hypertension (GH) was defined according to standard diagnostic criteria as new-onset systolic blood pressure ≥ 140 mmHg and/or diastolic blood pressure ≥ 90 mmHg, measured on at least two separate occasions at least 4 h apart, developing after 20 weeks of gestation in previously normotensive women. None of the included patients developed proteinuria or other signs of preeclampsia. At the time of diagnosis, the hypertension was classified as mild-to-moderate, and importantly, from a clinical perspective, all patients in the study group received standard antihypertensive pharmacotherapy with methyldopa (Dopegyt) to tightly control blood pressure until delivery.

The control group (N = 62) consisted of healthy women of similar age who had a normal pregnancy and delivered healthy newborns. Of the 62 women in the control group, 33 underwent cesarean section (53.2%). In the study group with gestational hypertension (9 patients), cesarean section was performed in 5 women (55.6%). Detailed clinical data presented in Table 4, including potential metabolic comorbidities, were rigorously documented for subsequent inclusion in multivariate statistical models.

### 4.2. Isolation of HM-MSCs and Immunophenotypic Assessment

Milk samples were transported to the Department of Clinical Genetics, Medical University of Lublin. The cellular fraction was isolated from whole HM by centrifugation. The cellular fraction containing HM-MSCs was isolated according to the method described by Hassiotou et al. [42,43]. Briefly, milk samples were centrifuged for 20 min at 805× *g*. The resulting cell pellet was washed twice with Phosphate-Buffered Saline (PBS) (Biomed, Lublin, Poland). This procedure yielded a heterogeneous cell fraction containing the HM-MSC population.

The isolated cells were used to establish short-term cell cultures. Cultures were maintained at 37 °C, 15% O_2_, 5% CO_2_, and 95% humidity. The culture medium consisted of Dulbecco’s Modified Eagle’s Medium (DMEM F-12 Corning, Manassas, VA, USA) supplemented with 10% Fetal Bovine Serum (FBS, ATCC, Teddington, UK) and 1% antibiotic (Sigma-Aldrich, Jerusalem, Israel). Details of the reagents and procedures used are presented in [23,38,44].

The presence of MSCs was confirmed by assessing the surface antigens: CD73, CD90, CD105, and CD146, and by excluding the hematopoietic markers: CD45, CD31, CD19, and CD14, using a Navios flow cytometer (Beckman Coulter, Bangalore, Karnataka, India), according to the protocol described previously [23,42]. Additionally, the morphology of the obtained cells was assessed using an IX81 microscope (Olympus, Tokyo, Japan) at 200× magnification, using the Xcellence RT system (Olympus, Tokyo, Japan).

### 4.3. RNA Isolation, Assessment of TSG-6 Expression, and MicroRNA Analysis

Total RNA was isolated from the heterogeneous human milk cellular fraction using a modified Chomczyński and Sacchi method, described in detail in [42,45,46]. Reverse transcription was then performed to synthesize cDNA molecules, which were used to assess *TSG-6* expression by qPCR. Expression levels were calculated using the RQ = 2^−DeltaDelta CT^ method [47]. Detailed methodological information is presented in [42,46,48].

The TaqMan probe Hs00200180_m1 targeting the *TSG-6* gene (NM_007115.3) was used for the PCR reaction. The reference gene was Glyceraldehyde 3-Phosphate Dehydrogenase (*GAPDH*) (NM_001289746.1; Hs99999905_m1).

In this study, we also analyzed the correlation of *TSG-6* gene expression with the levels of the miRs (miR-21-3p, miR-126-3p, and miR-155-5p). Data on the expression of these miRs were determined as part of a previously published research project on the same cohort of patients, and detailed procedures for miR isolation and analysis are described in the publication by Kondracka et al. [24].

### 4.4. Assessment of TSG-6 Protein Concentration in Whole Milk by ELISA

Whole milk samples were diluted 1:2 with nuclease-free water before analysis. To minimize matrix interference from the complex lipid and protein components of whole milk, the optimal 1:2 dilution was established following preliminary tests for linearity of dilution. Furthermore, intra-assay and inter-assay coefficients of variation (CV) were maintained within the manufacturer’s accepted limits. TSG-6 concentration was measured using the Human Tumor Necrosis Factor-inducible Gene 6 Protein ELISA Kit (cat. no. MBS7254543, MyBioSource, San Diego, CA, USA) according to the manufacturer’s instructions. All samples were analyzed in duplicate. Optical density was measured at 450 nm using an ETI MAX 3000 microplate reader (DiaSorin, Saluggia, Italy). TSG-6 concentrations were determined from a standard curve generated using a four-parameter logistic curve fit.

### 4.5. Statistical Analysis

Statistical analysis of the collected data was performed using Statistica 13 software (StatSoft, Inc., Tulsa, OK, USA). First, the distribution of quantitative variables was assessed using the Shapiro–Wilk test, which indicated conformity of the studied parameters with a normal distribution, allowing the use of parametric statistical tests. To evaluate the significance of differences in mean parameter values between the study groups (women with hypertension vs. the control group), the independent samples *t*-Student test was applied. Correlations between selected clinical parameters and *TSG-6* gene expression or protein levels were assessed using Pearson’s correlation test. To address the risk of false-positive findings arising from multiple comparisons (Table 3), the Benjamini–Hochberg False Discovery Rate (FDR) correction was applied. Adjusted *p*-values (q-values) were calculated simultaneously across all 25 tested correlations to rigorously control for the overall false discovery rate.

To determine the independent effect of gestational hypertension on *TSG-6* expression and protein concentration, comprehensive multivariable GLMs were constructed. To address potential confounders and baseline differences between groups, the models were adjusted for maternal age, pre-pregnancy Body Mass Index (BMI), gestational age (GA), and the presence of gestational diabetes. Downstream variables (such as neonatal acid–base parameters) were excluded from the primary GLM to prevent over-adjustment. Effect sizes were reported using partial eta-squared (ηp^2^), and model robustness was evaluated using the adjusted R^2^ statistic. For categorical variables (e.g., gestational hypertension), the healthy normotensive group served as the reference category.

## 5. Conclusions

In summary, this study demonstrates that maternal gestational hypertension is a significant factor associated with a reduction in *TSG-6* gene expression in the heterogeneous milk cellular fraction containing HM-MSCs and TSG-6 protein concentration in early human milk, irrespective of maternal age, pre-pregnancy BMI, gestational age, and gestational diabetes. Furthermore, our exploratory analyses suggested that, under healthy physiological conditions, TSG-6 levels in human milk were positively correlated with parameters reflecting the metabolic and acid–base status of the newborn, including cord blood pH.

Multivariate GLM analysis confirmed that the absolute decrease in anti-inflammatory TSG-6 is associated with the gestational hypertension phenotype, independent of metabolic diseases or preterm birth. However, given that all participants with hypertension received methyldopa, the independent contributions of vascular pathology and antihypertensive treatment cannot be distinguished. Furthermore, the concomitant reduction in *TSG-6* expression and its closely correlated signature of immunomodulatory microRNAs (including miR-155 and miR-21) indicates a potential alteration in the trophic profile of colostrum cells.

We hypothesize that this reduction in the cytoprotective properties of human milk may compromise the support provided to the gastrointestinal and immune systems of newborns at greatest risk of complications. However, further longitudinal and functional studies are needed to determine the actual impact of reduced TSG-6 levels in human milk on intestinal barrier integrity and clinical outcomes in newborns.

To the best of our knowledge, this is the first study to demonstrate an association between maternal gestational hypertension and the TSG-6 anti-inflammatory axis in early human milk. Expanding this line of research within the DOHaD framework may provide further insight into how perinatal stem cells and human milk contribute to the development of the newborn’s immune system and how maternal pathologies may affect this critical mother–infant interface.

## Figures and Tables

**Figure 1:**
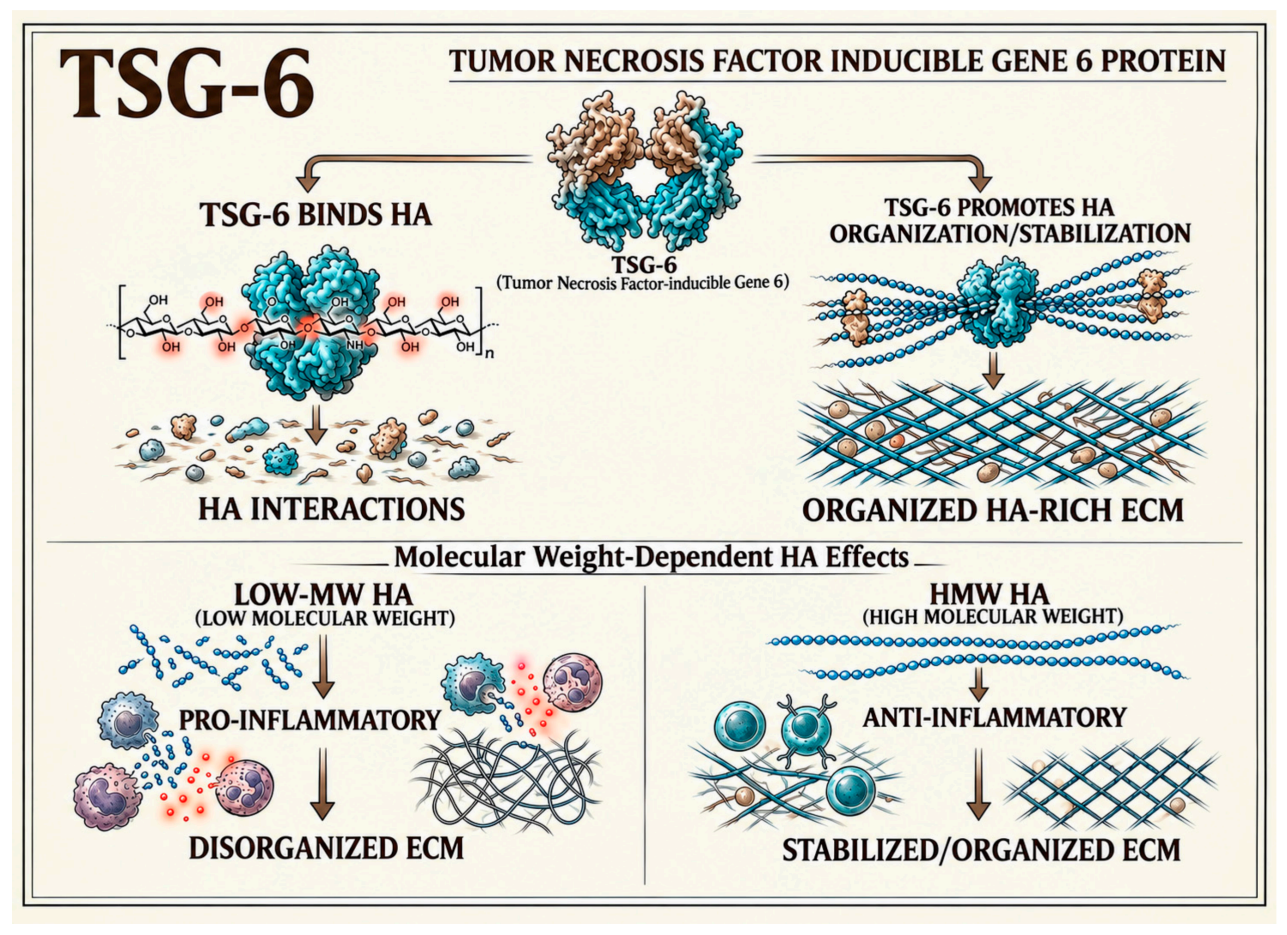
Mechanism of action of the TSG-6 protein, mediated by HA. Illustration generated using AI (Gemini 1.5 Pro, Google, Mountain View, CA, USA) based on detailed prompts provided by the authors.

**Figure 2:**
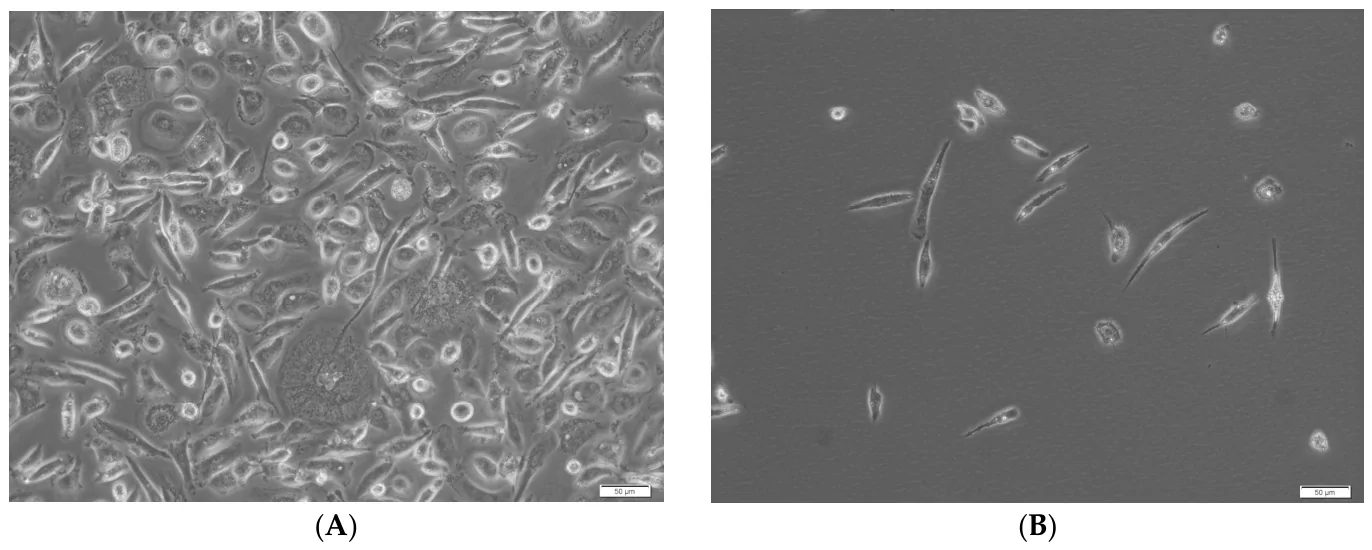
Morphology of cells derived from human milk (HM) in in vitro culture. (**A**) A heterogeneous cell population observed immediately after culture establishment. (**B**) Adherent cells exhibiting a characteristic mesenchymal, spindle-shaped morphology after 48 h of culture. Images were captured at 200× magnification using bright-field microscopy (Xcellence RT system with an IX81 inverted microscope, Olympus).

**Figure 3:**
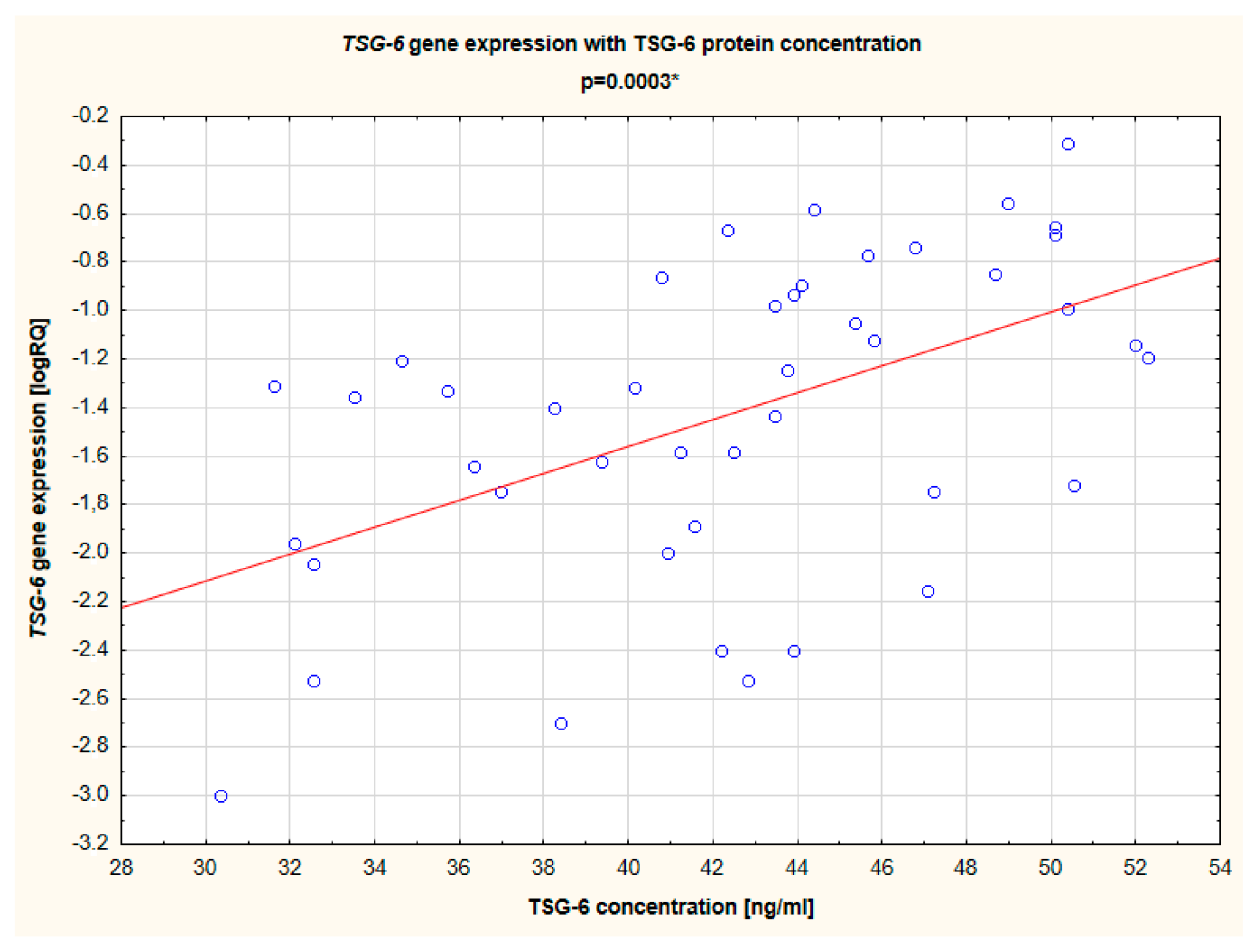
Scatter plot: TSG-6 protein concentration [ng/mL] in relation to *TSG-6* gene expression [logRQ], correlation coefficient r = 0.479, *p* < 0.05. Pearson correlation. The red line indicates the linear trend line. * *p* < 0.05 indicates statistical significance.

**Figure 4:**
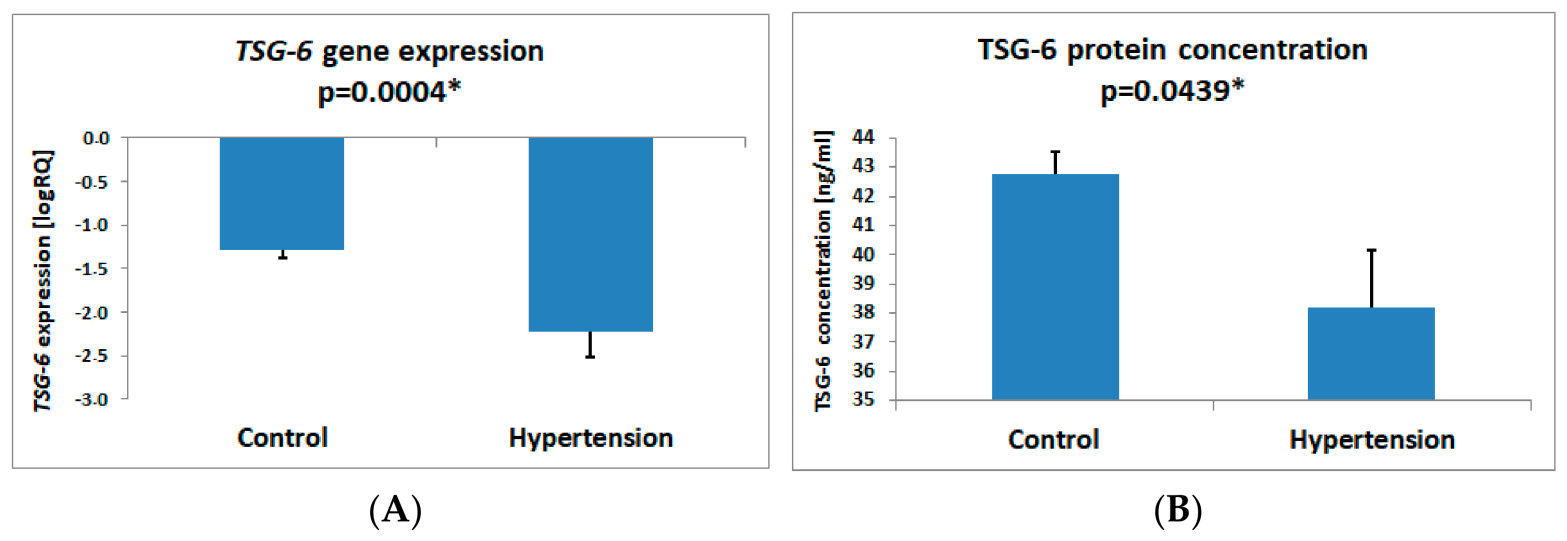
(**A**) *TSG-6* gene expression in the human milk cellular fraction in relation to the presence of maternal gestational hypertension. (**B**) TSG-6 protein concentration in HM in relation to the presence of maternal gestational hypertension. * *p* < 0.05.

**Figure 5:**
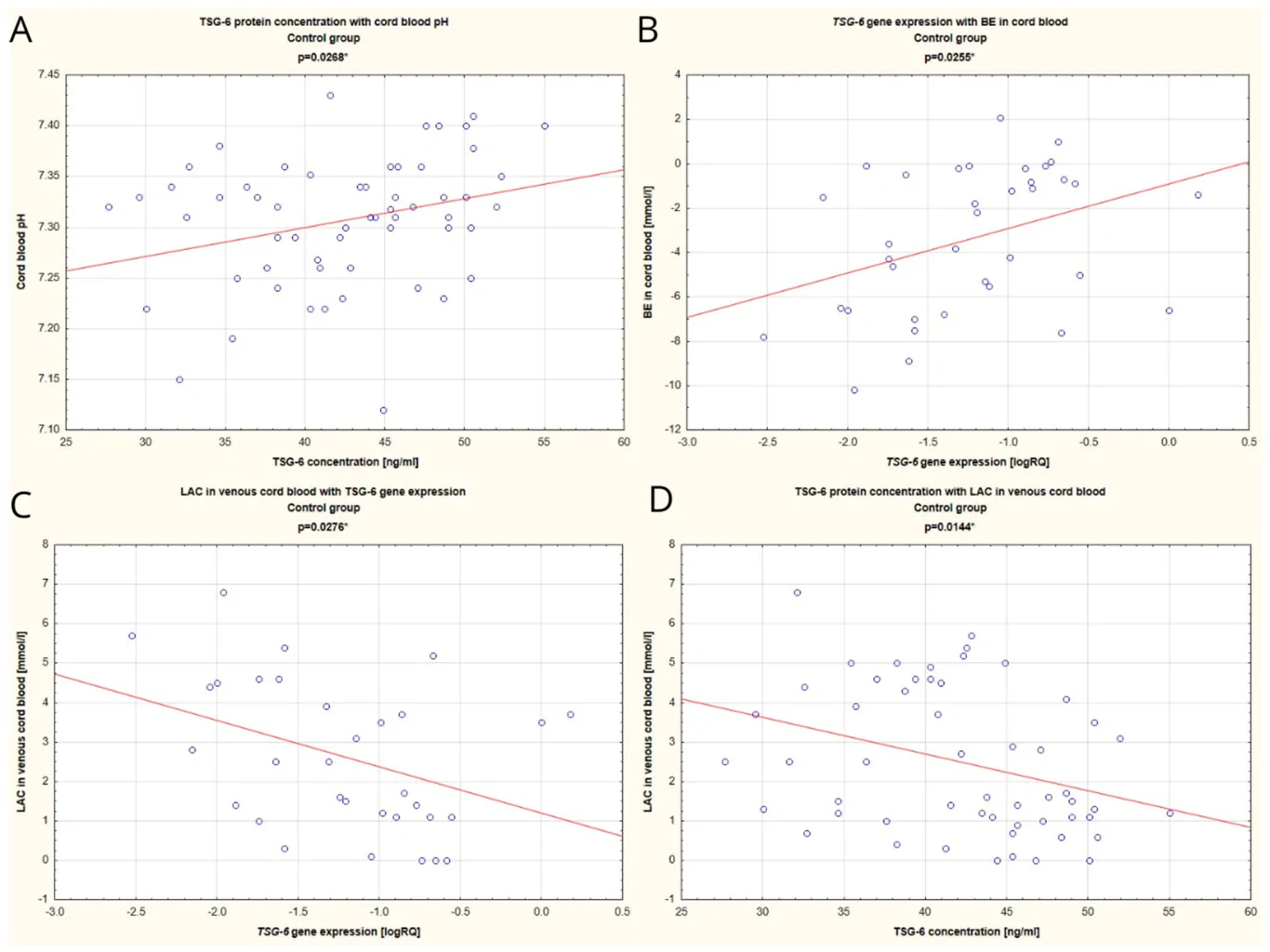
Plot of associations observed in the control group between (**A**) TSG-6 protein concentration in HM and umbilical cord blood pH (r = 0.481 *p* < 0.05), (**B**) logRQ *TSG-6* in the HM cellular fraction and umbilical cord blood BE (r = 0.467 *p* < 0.05), (**C**) logRQ *TSG-6* in the HM cellular fraction and venous umbilical cord blood LAC (r = −0.529 *p* < 0.05), (**D**) TSG-6 protein concentration in HM and venous umbilical cord blood LAC (r = −0.387 *p* < 0.05). These associations did not remain statistically significant after FDR correction (q > 0.05) and should be interpreted strictly as exploratory, hypothesis-generating observations. The red line indicates the linear trend line. * *p* < 0.05 indicates statistical significance.

**Figure 6:**
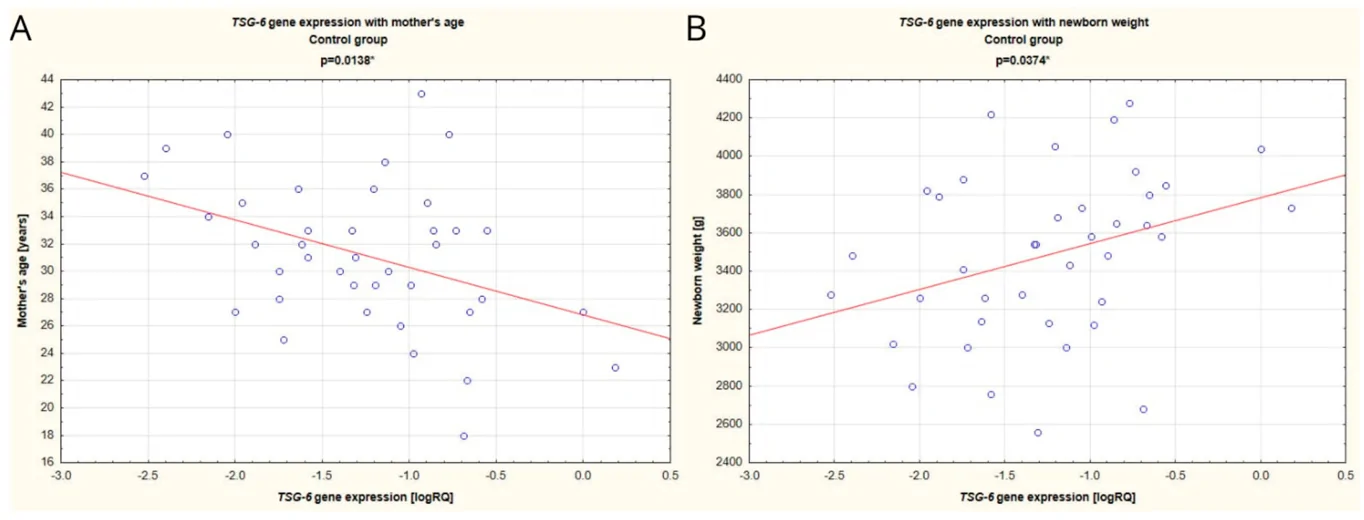
Plot of associations observed in the control group between (**A**) logRQ *TSG-6* in the HM cellular fraction and maternal age (r = −0.391 *p* < 0.05). (**B**) logRQ *TSG-6* in the HM cellular fraction and newborn’s body weight (r = 0.385 *p* < 0.05). These associations did not remain statistically significant after FDR correction (q > 0.05) and should be interpreted strictly as exploratory, hypothesis-generating observations. The red line indicates the linear trend line. * *p* < 0.05 indicates statistical significance.

**Figure 7:**
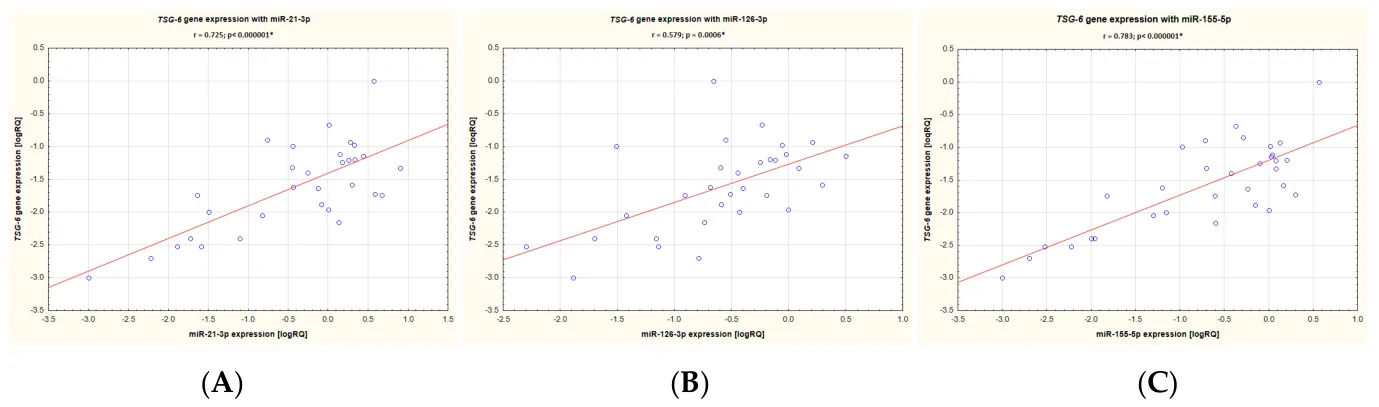
Plot of associations observed in the control group between (**A**) logRQ *TSG-6* in the HM cellular fraction and logRQ miR-21-3p (r = 0.725 *p* < 0.05). (**B**) logRQ *TSG-6* in the HM cellular fraction and miR-126-3p (r = 0.579 *p* < 0.05). (**C**) logRQ *TSG-6* in the HM cellular fraction and miR-155-5p (r = 0.783, *p* < 0.05). The red line indicates the linear trend line. * *p* < 0.05 indicates statistical significance.

**Table 1:** *TSG-6* gene expression and TSG-6 protein concentration in HM in relation to maternal gestational hypertension. * *p* < 0.05 *t*-Student test.

Parameter	Control			Gestational Hypertension			*p*-Value
	N	Mean	SD	N	Mean	SD	
*TSG-6* gene expression [logRQ]	39	−1.293	0.542	9	−2.235	0.682	0.0004 *
TSG-6 protein concentration [ng/mL]	62	42.738	6.151	9	38.208	5.887	0.0439 *

Bold font indicates statistically significant values (*p* < 0.05).

**Table 2:** Multivariable General Linear Model (GLM) predicting *TSG-6* gene expression and protein concentration in early human milk.

Predictors	F-Value	*p*-Value	Effect Size (ηp^2^)	Power
Model 1: *TSG-6* gene expression [logRQ]				
Maternal Age (years)	2.004	0.165	0.051	0.281
Pre-pregnancy BMI	0.391	0.535	0.011	0.094
Gestational Age (weeks)	0.272	0.605	0.007	0.080
Gestational Diabetes	0.354	0.556	0.010	0.089
Gestational Hypertension	7.337	0.010 *	0.166	0.751
Model 2: TSG-6 protein concentration [ng/mL]				
Maternal Age (years)	3.063	0.085	0.046	0.407
Pre-pregnancy BMI	0.368	0.546	0.006	0.092
Gestational Age (weeks)	0.211	0.647	0.003	0.074
Gestational Diabetes	0.697	0.407	0.011	0.130
Gestational Hypertension	4.276	0.043 *	0.063	0.531

Statistically significant independent predictors (* *p* < 0.05) are highlighted in bold. ηp^2^ represents partial eta-squared (effect size).

**Table 3:** Table presenting Pearson’s correlation coefficient (r) between the relative logRQ *TSG-6* and TSG-6 protein concentration [ng/mL] in early HM and parameters characterizing pregnancy, maternal health status, and neonatal health status. * *p* < 0.05 Pearson’s correlation test.

Variable	Control		Gestational Hypertension	
	*TSG-6* Gene Expression [logRQ]	TSG-6 Protein Concentration [ng/mL]	*TSG-6* Gene Expression [logRQ]	TSG-6 Protein Concentration [ng/mL]
GA	0.143	−0.190	0.082	0.381
Age	−0.391 *	−0.214	0.071	−0.115
Pre-pregnancy body mass	−0.043	0.215	−0.400	−0.215
Pre-pregnancy BMI	−0.106	0.141	−0.367	−0.185
CRP (mother)	−0.049	0.063	−0.220	−0.379
Glucose (mother)	−0.280	0.186	0.526	0.229
WBC (mother)	−0.027	−0.048	−0.029	−0.225
HB (mother)	0.182	0.021	−0.312	0.085
PLT (mother)	0.205	0.168	−0.370	−0.230
CRP (Child)	−0.006	−0.052	0.034	−0.182
Glucose (Child)	0.088	0.192	0.408	0.017
Total bilirubin (Child)	0.033	0.181	0.086	−0.125
HB (Child)	−0.093	0.048	−0.074	−0.373
WBC (Child)	0.024	−0.002	−0.426	0.052
PLT (Child)	0.212	0.038	−0.342	−0.022
Apgar scale	−0.029	−0.061	−0.683	0.233
Length [cm]	0.117	−0.021	0.002	0.157
Newborn’s body weight [g]	0.385 *	−0.059	−0.075	0.170
pH	0.219	0.481 *	0.195	0.265
BE	0.467 *	0.190	−0.036	0.275
Arterial LAC	−0.486	−0.350	−0.088	−0.283
Venous LAC	−0.529 *	−0.387 *	−0.417	−0.182
logRQ miR-21-3p	0.725 **	−0.021	0.733	0.299
logRQ miR-126-3p	0.579 **	0.043	0.155	0.251
logRQ miR-155-5p	0.783 **	0.062	0.775	0.171

* *p* < 0.05 (Nominal significance/Exploratory analyses): Statistically significant based on unadjusted Pearson’s correlation test. Following Benjamini–Hochberg False Discovery Rate (FDR) correction for multiple testing, these associations did not maintain statistical significance (q > 0.05) and should be interpreted as exploratory observations. ** q < 0.05 (FDR-adjusted significance): Highly statistically significant correlations that robustly survived the Benjamini–Hochberg FDR correction for multiple comparisons. Abbreviations: GA—gestational age, BMI—body mass index, CRP—C-reactive protein, WBC—white blood cells, HB—hemoglobin, PLT—platelets, BE—base excess, LAC—lactate.

**Table 4:** Characteristics of the control and study group.

Parameter	Control			Gestational Hypertension			*p*-Value
	N	Mean	SD	N	Mean	SD	
Number of pregnancies	62	2.175	1.351	9	1.778	1.093	0.403
GA [week]		38.937	1.378		37.333	2.598	0.005 *
Maternal age [years]		31.016	5.443		30.556	1.810	0.803
Pre-pregnancy body mass [kg]		63.326	10.250		67.278	9.438	0.279
Pre-pregnancy BMI		22.760	3.243		25.302	3.838	0.035 *
CRP (Mother) [mg/L]	62	4.821	5.790	9	15.860	27.535	0.052
Glucose (Mother) [mg/dL]		85.366	11.979		83.556	7.367	0.666
WBC (Mother) [10^9^/L]		11.119	2.722		10.012	1.667	0.241
HB (Mother) [g/dL]		12.395	0.936		11.725	0.987	0.062
PLT (Mother) [10^9^/L]		226.581	59.097		217.444	61.924	0.668
CRP (Child) [mg/L]	62	5.725	8.928	9	2.078	1.110	0.228
Glucose (Child) [mg/dL]		61.253	14.895		65.911	14.972	0.384
Total bilirubin (Child) [mg/dL]		8.316	3.321		10.830	3.097	0.036 *
WBC (Child) [10^9^/L]		15.061	6.040		11.649	3.898	0.105
HB (Child) [g/dL]		17.275	1.953		19.867	2.576	0.001 *
PLT (Child) [10^9^/L]		277.968	59.079		237.111	63.349	0.058
Apgar scale		9.937	0.304		9.111	1.167	0.00001 *
Newborn’s body weight [g]		3467.94	445.523		2905.556	811.774	0.002 *
Newborn’s body length [cm]		53.698	2.637		50.444	5.364	0.004 *
pH	59	7.306	0.065	9	7.308	0.075	0.923
BE [mmol/L]		−3.134	3.184		−3.622	2.108	0.659
Arterial LAC [mmol/L]		3.571	1.924		3.367	2.257	0.772
Venous LAC [mmol/L]		2.461	1.800		2.178	1.871	0.665

* *p* < 0.05 *t*-Student test, Abbreviations: GA—gestational age, BMI—body mass index, CRP—C-reactive protein, WBC—white blood cells, HB—hemoglobin, PLT—platelets, BE—base excess, LAC—lactate.

## Data Availability

The raw data supporting the conclusions of this article will be made available by the authors upon request.

## Supplementary Materials

The following supporting information can be downloaded at https://www.mdpi.com/article/10.3390/ijms27198573/s1.
