# Supplementary material for: Maternal Gestational Hypertension Is Associated with Reduced TSG-6 in Human Milk: Potential Implications for Neonatal Immunological Support
Source: Int J Mol Sci. 2026 Sep 25;27(19):8573. doi: 10.3390/ijms27198573 (PMC13665462; doi:10.3390/ijms27198573)
Supplement: Supplementary file 1 [file ijms-27-08573-s001.zip › ijms-4557855-supplementary.pdf]

Maternal Gestational Hypertension Is Associated with Reduced TSG-6 in Human Milk: Potential Implications for Neonatal Immunological Support

Supplementary methods: Flow cytometry analysis

Immunophenotypic characterization of the isolated human milk cell fraction was performed using a Navios flow cytometer (Beckman Coulter). Cells were stained using the DuraClone MSC marker panel (Beckman Coulter, Brea, CA, USA) in accordance with the manufacturer's instructions and our previously established, standardized laboratory protocols.

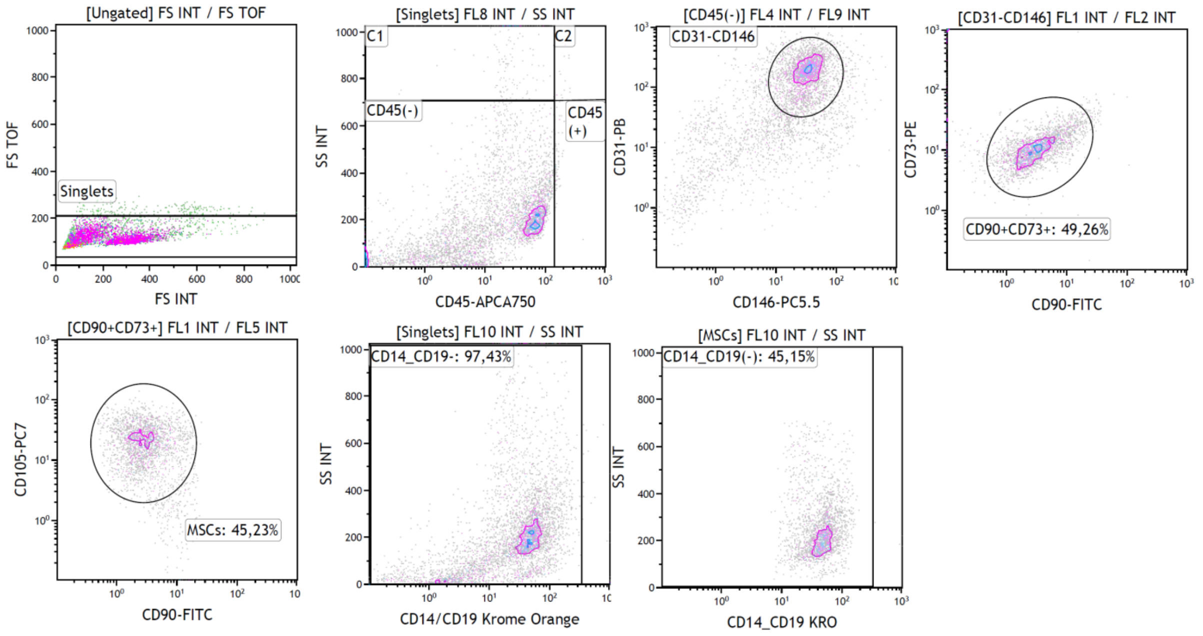

Supplementary Figure S1. Flow cytometry gating strategy for the immunophenotypic characterization of human milk-derived mesenchymal stem cells (HM-MSCs).

Representative flow cytometry dot plots illustrating the identification of HM-MSC subpopulations within the heterogeneous cell fraction of early human milk. First, single cells (singlets) were isolated based on the relationship between forward scatter intensity (FS INT) and time of flight (FS TOF) to exclude doublets. Next, hematopoietic cells were excluded by selecting the CD45(-) population. Subsequent gating steps confirmed the absence of the endothelial marker CD31 and the presence of the mesenchymal marker CD146. The final target population was identified based on the distinct co-expression of canonical MSC surface antigens: CD90, CD73, and CD105. Additional verification confirmed the absence of monocyte/B-lymphocyte marker expression (negative for CD14/CD19) in the selected cell population. The identified HM-MSCs accounted for approximately 45% of the events (cells) analyzed in this representative sample.
